# Non-Coding RNAs in Cell-to-Cell Communication: Exploiting Physiological Mechanisms as Therapeutic Targets in Cardiovascular Pathologies

**DOI:** 10.3390/ijms24032205

**Published:** 2023-01-22

**Authors:** Javier Laura Francés, Elettra Musolino, Roberto Papait, Christina Pagiatakis

**Affiliations:** 1 IRCCS Humanitas Research Hospital, 20089 Rozzano, Italy; 2 Department of Biotechnology and Life Sciences, University of Insubria, 21100 Varese, Italy

**Keywords:** cardiovascular disease, ncRNAs, epigenetics, extracellular vesicles, nanoparticles

## Abstract

Cardiovascular disease, the leading cause of death worldwide, has been characterized at the molecular level by alterations in gene expression that contribute to the etiology of the disease. Such alterations have been shown to play a critical role in the development of atherosclerosis, cardiac remodeling, and age-related heart failure. Although much is now known about the cellular and molecular mechanisms in this context, the role of epigenetics in the onset of cardiovascular disease remains unclear. Epigenetics, a complex network of mechanisms that regulate gene expression independently of changes to the DNA sequence, has been highly implicated in the loss of homeostasis and the aberrant activation of a myriad of cellular pathways. More specifically, non-coding RNAs have been gaining much attention as epigenetic regulators of various pathologies. In this review, we will provide an overview of the ncRNAs involved in cell-to-cell communication in cardiovascular disease, namely atherosclerosis, cardiac remodeling, and cardiac ageing, and the potential use of epigenetic drugs as novel therapeutic targets.

## 1. Introduction

Cardiovascular diseases (CVD) are the leading cause of death worldwide, and their incidence continues to increase as a result of changes in lifestyle and the increase in average lifespan. Previous studies reported that in Europe alone around 4 million people die of CVD every year, accounting for 44% of all deaths [1,2].

Although many studies over the last several decades have elucidated several molecular mechanisms involved in the maintenance of cardiovascular homeostasis and their involvement in pathology upon dysregulation, the role of epigenetic mechanisms in such contexts still remain widely unknown. A prominent class of epigenetic mechanisms is that of non-coding RNAs, which have become increasingly important in the study of cardiac homeostasis and cardiovascular disease. Since more than 97% of the genome is untranslated, understanding the mechanisms by which the non-coding transcriptome exerts its function on pathogenesis is imperative for the development of novel therapeutic targets [3].

Non-coding RNAs (ncRNAs) comprise the majority of our genome and are not translated into proteins, thus accounting for a myriad of regulatory mechanisms and pathways at the level of the epigenome. Non-coding RNAs are generally classified based on their length (short and long). Short ncRNAs include RNA molecules containing less than 200 nucleotides, including PIWI-interacting RNAs, small interfering RNAs (siRNAs), and microRNAs (miRNAs), and are generally highly conserved. On the other hand, long-ncRNAs (lncRNAs) are longer than 200 nucleotides, and are poorly conserved at the level of their primary sequence. MicroRNAs inhibit gene expression by binding to the 3′-UTR (untranslated region) of their target mRNAs, resulting in a degradation of the target mRNA and a subsequent inhibition of protein translation, a mechanism of action which is very well conserved [4]. On the other hand, the heterogeneous long non-coding RNAs can regulate gene expression at the transcriptional and translational levels, and function through a plethora of different mechanisms. Their function is usually linked to their genomic position and orientation with respect to their neighboring protein-coding gene, and has been shown to have high cell and tissue specificity [5].

**Figure 1:**
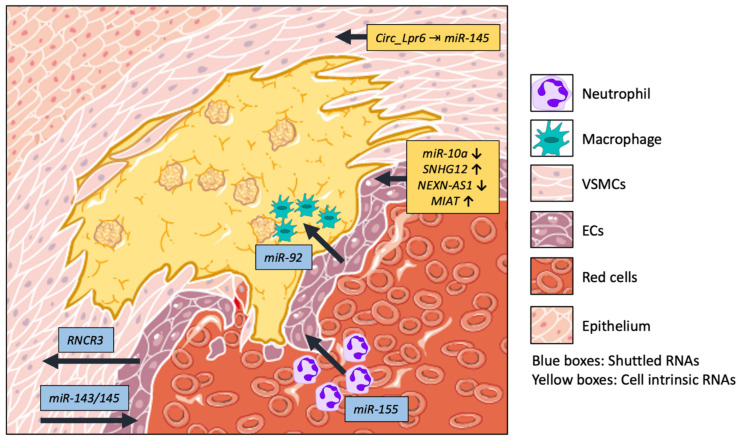
Summary of non-coding RNAs involved in atherosclerosis.

**Figure 2:**
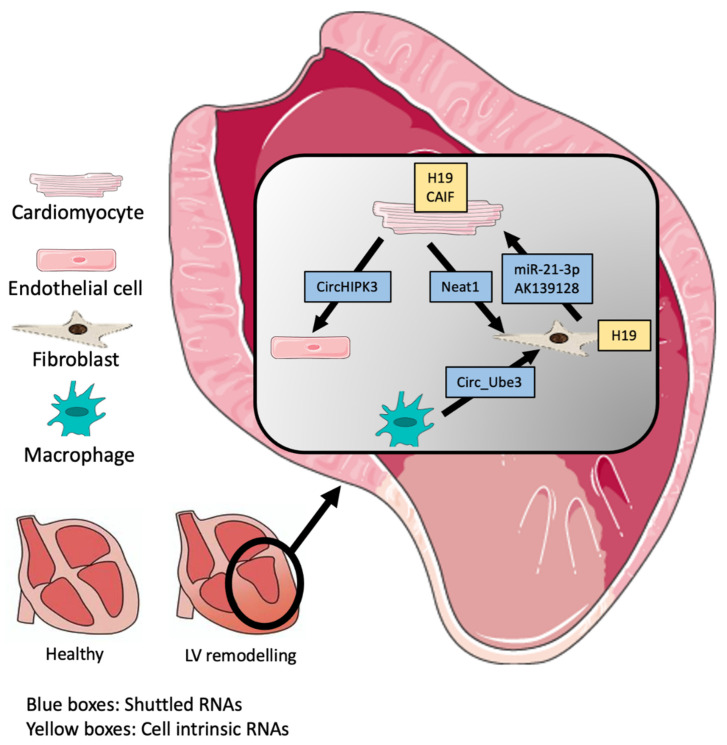
Summary of non-coding RNAs involved in cardiac remodeling.

Several ncRNAs have been implicated in the regulation of key pathways and enzymes involved in various pathophysiologies, and could therefore be a promising target for epigenetic and genetic regulation and pharmacological targeting. In this review, we will outline the role of non-coding RNAs in atherosclerosis and cardiac remodeling, describing the roles of these transcripts in mediating homeostasis and disease by means of cellular communication, and how such mechanisms can be exploited for the discovery of novel therapeutic targets (Summarized in Figure 1 and Figure 2).

## 2. Atherosclerosis

Atherosclerosis is an increasingly common CVD and the principal cause of myocardial infarct (MI) and stroke in Western countries. Risk factors associated with this disease include ageing, hyperlipidemia, obesity, diabetes, hypertension, and smoking, to name a few. The origins of this disease comprise lipid accumulation and fibrous tissue generation in the arteries and vessels that can form an atherosclerotic plaque. The eventual rupture of atherosclerotic plaques often leads to thromboembolic events [6].

At the cellular level, there is an orchestration of events between different cell types in response to endothelial cell (EC) dysfunction. This dysfunction results from EC replacement by proliferative ECs that eventually develops into apoptosis and necrosis when the disease becomes aggravated. Following EC injury, inflammatory cells infiltrate the vascular smooth muscle cells (VSMCs) which become activated from a quiescent to a proliferative state, intensifying atherosclerotic plaque formation [7].

There is solid evidence that ncRNAs play a central role in the progression of atherosclerosis, not only by participating in molecular and signaling mechanisms, but also by mediating cell-to-cell communication such as through extracellular vesicles (EVs) or tunneling nanotubes [8,9]. Non-coding RNAs are transcripts without protein-coding potential that account for 97% of the transcribed genome. Such transcripts are classified by their length (bp) or secondary structure and mechanism of function: miRNAs (≈20 nt), long non-coding RNAs (lncRNA > 200 bp), and circular RNAs, to name a few [10].

Current therapies for atherosclerosis include invasive surgical procedures or medications that impede the accumulation of fatty deposits; however, such strategies do not fully prevent the evolution of the disease [11]. Thus, understanding the mechanisms by which ncRNAs affect atherosclerotic plaque formation is of key importance. The mechanisms by which ncRNAs regulate pathophysiology still remain elusive in the context of cardiovascular disease; however, exploiting these molecules could provide a new source of feasible targets for drug discovery. Several ncRNAs have been described in the context of atherosclerosis, specifically for mediating cellular communication (Table 1). Aberrant communication between the cell types that comprise an organ could be detrimental for the maintenance of homeostasis, and they are therefore important molecular targets for understanding the pathophysiology of cardiovascular disease (Figure 1).

### 2.1. ncRNAs Mediating Cell Communication in Atherosclerosis

Cross-talk between the various cell types of an organ is necessary for the maintenance of homeostasis. Communication between ECs and VSMCs has been a considerable area of study with respect to atherosclerosis over the last decade, and ncRNAs have been highly implicated in the formation of atherosclerotic plaques [8].

The well-studied miR-143/145 cluster has been shown to be dysregulated in atherosclerosis in human aortas and mice models. The loss of miR-143/145 in mice activates VSMCs and they switch from a quiescent, differentiated state to a proliferative one, inducing structural modifications in the aorta. However, in ECs, miR-143/145 modulates the response to shear stress [20]. Interestingly, when VSMCs and ECs are in contact, miR-143/145 was shown to translocate from VSMCs to ECs via membrane protrusions named tunneling nanotubes in co-cultures and in intact vessels. This transfer is mediated by the transforming growth factor β (TGFβ) pathway [9], a major regulator of VSMC differentiation and vessel stabilization [21,22]. Interestingly, TGFβ silencing suppresses miR-143/145 passage. Once present in ECs, the miR143/145 cluster reduces its angiogenic potential by inhibiting two essential genes for angiogenesis, namely *Hexokinase II* (*HKII*) and *Integrin* β *8* (*ITG*β*8*) [9]. Therefore, this cluster could be of critical importance in the maintenance of homeostasis of VSMCs and ECs, thus preventing aberrant activation of these cell types and preventing atherosclerotic plaque formation.

A novel pathway described by K Shan et al. demonstrated the atheroprotective role of the lncRNA *RNCR3* in mice and humans. This lncRNA is upregulated and released from ECs in aortic atherosclerotic lesions, and acts as a competing endogenous RNA (ceRNA), regulating miR-185-5p in the recipient cells (VSMCs) where it forms a feedback loop with Kruppel-like factor 2 (Klf2) to regulate cell function. Intriguingly, miR-185-5p mediates the interaction between *RNCR3* and Klf2 [12], a known transcriptional factor that confers vasoprotection on ECs [23]. The lncRNA *RNCR3* decreases the targeting concentration of miR-185-5p depressing *KLF2*-mRNA levels [12]. In vivo knockdown of *RNCR3* results in an aggravated development of atherosclerosis, together with hypercholesterolemia and inflammation [12]. Therefore, it is of importance to note that communication between cells through extracellular vesicles, specifically in the transport of ncRNAs, is a key player in the onset of disease and should therefore be considered as a strong therapeutic target.

Despite the relevance of the inflammatory response on atherosclerotic plaque formation, the focus of these mechanisms has been mostly focused on the EC–VSMC axis [24]. However, it has been shown that ECs export miR-92 in EVs to target macrophages upon plaque formation in aortas [25]. Macrophages are the first immune cells present in the atherosclerotic plaque, and are responsible for disease progression. Co-culture of ECs and macrophages, together with gain- and loss-of-function studies of miR-92, demonstrated that upon miR-92 intake, macrophages switch their phenotype to pro-atherosclerotic, through the inhibition of Klf4 (*Krüppel-like factor 4*) [13], a transcription factor related to macrophage plasticity [26]. This study revealed that the uptake of miR-92 by macrophages drives them to a pro-atherosclerotic phenotype and highlights miR-92 as a potential target for regulating vascular diseases.

Moreover, miR-155 links neutrophils with atherosclerosis, notwithstanding their absence within the atherosclerotic plaque. Studies using cultured human coronary endothelial cells with neutrophil-derived vesicles from miR-155^-/-^ mice demonstrated that neutrophils promote detrimental vascular inflammatory gene expression by delivering miR-155 to arterial ECs in microvesicles (MVs), enhancing NF-κB activation. This activation results in the release of pro-inflammatory molecules that aggravate atherogenesis [14].

### 2.2. ncRNAs Mediating Cell Pathophysiology 

ncRNAs have been shown to be important in the maintenance of homeostasis, regulating a myriad of processes within the cell. The aberrant regulation of ncRNAs has been a very important topic in the understanding of the development, cellular function and, importantly, pathophysiology of many diseases.

An elegant paper by Hall et al. outlined the importance of a circRNA, named *Circ_Lpr6*, for vascular pathogenesis in humans and mice. RNA sequencing coupled with bioinformatic analysis revealed this novel circRNA, which possesses miRNA binding sites conserved between mice and humans in VSMCs. *Circ_Lpr6* acts as a sponge for the above-mentioned miR-145, thus regulating its downstream targets. Gain- and loss-of-function studies demonstrated that upon wound-healing stress, *Circ_Lpr6* hinders the miR-145-mediated regulation of VSMC differentiation, migration, and proliferation. Furthermore, in vivo overexpression of *Circ_Lpr6* in mice resulted in a decreased neointimal area in aortas. *Circ_Lrp6* is necessary for the counterbalancing of miR-145 in VSMCs, providing additional evidence that ncRNAs are an important target in this disease, as the ratio of *Circ_Lpr6* bound to miR-145 versus unbound plays a role in vascular pathogenesis [15].

Ding-Yu Lee et al. used an in vitro flow system, and the well-stablished *ApoE*^-/-^ rat model of atherosclerosis to demonstrate that downregulating miR-10a in both aortic endothelium and blood serum is highly associated with atherogenesis. Upon induction, miR-10a reduces the atherosclerotic lesion in the atherosclerotic vessel wall by inhibiting vascular cell adhesion signaling pathways, namely GATA6/VCAM-1. Downregulation of GATA6/VCAM-1 results in a reduction in leukocyte infiltration, thus decreasing plaque formation. Therefore, this study shows that the downregulation of miR-10a is associated with atherosclerosis, and its induction could prove to be a novel therapeutic target for this disease [16].

It is well known that ROS promote oxidative stress and DNA damage, which amongst other effects, leads to EC dysfunction [27]. The link between DNA damage and the progression of atherosclerotic plaque formation by promoting cell senescence and apoptosis has been previously described [28]. In this context, it was shown that the lncRNA *SNHG12* is upregulated in atherosclerotic plaques of aortas of mice, pigs, and humans. LC-MS/MS (Liquid chromatography tandem mass spectrometry) analysis identified DNA-PK (DNA-dependent protein kinase) as the target of *SNHG12* [17]. This protein is an important sensor and mediator of DNA damage [29]. *SNHG12* knockdown using a GapmeR approach inhibits the interaction between DNA-PK and Ku70/Ku80, two proteins that bind double strand breaks and mediate non-homologous repair, thus exacerbating DNA damage and aggravating EC injury upon atherosclerotic plaque formation [17]. This study provides yet another example of the importance of ncRNAs in mediating pathogenesis.

Another novel lncRNA reported in atherosclerotic plaque formation is *NEXN-AS1*. Hu et al. revealed that *NEXN-AS1* is downregulated in the ECs of human and mouse plaques during the development of atherosclerosis. This lncRNA is transcribed in an antisense manner with respect to its target gene, *NEXN* (nexilin F-Actin binding protein), which is important in cardiac T-tubule formation: cardiac loss of *NEXN* in mice resulted in a rapid progression to dilated cardiomyopathy [30]. *NEXN-AS1* interacts with the chromatin remodeler BAZ1A to regulate *NEXN* expression. More specifically, BAZ1A promotes chromatin condensation in the *NEXN* locus in response to atherosclerotic plaque formation. *NEXN-AS1* reduces BAZ1A activity to trigger chromatin relaxation, thus leading to *NEXN* expression. This increase in expression is beneficial against plaque formation in *ApoE*^–/–^ mice fed with a Western (high-fat) diet. In these mice, *NEXN* silencing aggravates atherosclerotic lesions while *NEXT* overexpression mitigates them by inhibiting EC activation and monocyte recruitment to the atherosclerotic plaque. This study therefore demonstrates the therapeutic potential of both *NEXN-AS1* and *NEXN* [18] in the regulation of atherosclerosis, providing yet another example of ncRNAs as potential druggable targets.

Myocardial Infarction Associated Transcript (*MIAT*) is a lncRNA detected in cohorts of patients with myocardial infarct [31]. Previously, *MIAT* was described as a sponge-lncRNA regulating miR-150 in different pathologies involving different cell types such as cardiomyocytes (CMCs) [32], ECs [33], and epithelial cells [34]. Recently, RNA profiling studies revealed an increase in *MIAT* in patients with advanced carotid plaques, as well as in murine and porcine carotid plaques, and in human carotid smooth muscle cells (hCSMC) [19]. Interestingly, GapmeR knockdown of *MIAT* reduced proliferation and increased apoptosis in hCSMC. In hCSMC, *MIAT* is mostly present in the nuclear fraction, and it affects SMC proliferation by regulating transcription factors comprising the ERK/ELK1/EGR1 pathway (extracellular signal-regulated kinase/ETS transcription factor ELK1/early growth response 1) [19]. These transcription factors have been described as activators of stress-responsive genes in atherosclerotic plaque formation, including PDGF and TGF-β [35]. Additionally, bioinformatic analysis and functional luciferase assays demonstrated the interaction between *MIAT* and the promoter of *KLF4*. KLF4 induces a phenotypic switch from SMC to macrophage-like cells, which has been shown to promote atherosclerosis progression [36]. This provides yet another example of an ncRNA that is necessary in regulating pathology onset, and could therefore be an important target for novel therapeutic strategies.

## 3. Pathological cardiac remodeling

Cardiac remodeling refers to the process by which the heart undergoes a rearrangement in size, shape, and function [37] as a result of molecular, metabolic, epigenetic and cellular changes. Pathological remodeling arises in response to a chronic maladaptive process derived from hypertrophy, MI, apoptosis, vascular dysfunction, necrosis, ventricular dilatation, myocarditis, or cardiac fibrosis [38]. Although the prognosis of these diseases is different, they all result in the process of cardiac remodeling, and at the cellular and epigenetic level, have many characteristics in common [39].

Therapeutic approaches against cardiac remodeling aim to restore the regeneration capability of the heart, and more specifically, cardiomyocytes. With no success in reducing mortality to date, these therapies include drug targeting, tissue engineering, and gene and cell therapies [40]. Only one drug-based RNA technology is currently in clinical trials [41]; however, this drug is based on mRNA delivery, and thus, newer RNA candidates are needed, specifically ncRNA. Due to their malleability, ncRNAs exhibit a therapeutic potential subjected to intense investigation over the last decade [42,43,44]. However, the implementation of lncRNAs as therapeutic targets requires further research. lncRNAs mediating cell communication and regulating cardiac remodeling present an unexplored source of potential targets (Figure 2 and Table 2).

### 3.1. ncRNAs Mediating Cell Communication in Cardiac Remodelling

Anselmo et al. recently identified a release of extra-cellular vesicles from CMCs under myocardial hypoxic stress. Through an exhaustive cytometry approach, they isolated EVs from the plasma of patients with different CVDs and from hypoxic hIPS-CMs. These vesicles were found to be enriched with miRNAs [54], indicating a cross-talk between the various cell-types of the heart, and more importantly, the transport of ncRNAs for the re-establishment of homeostasis post stress. Furthermore, the lncRNAs ENSMUST00000122745 and *Neat1* are released in small and large EVs, respectively, in response to hypoxic stimuli in HL-1 CMCs, and uptaken by fibroblasts, driving them to a pro-fibrotic phenotype. Silencing of *Neat1* in vitro induces apoptosis by targeting p53 downstream genes, which are cell-cycle regulators and promote cell survival. Instead, transient overexpression of ENSMUST00000122745 has no effect on fibroblast function, indicating that further research is needed for elucidation of the mechanism of action of this lncRNA [45].

On the other hand, miR-21-3p translocates from fibroblasts to cardiomyocytes in a paracrine fashion to induce cardiomyocyte hypertrophy in a murine model of pressure overload. Protein profiling identified that miR-21-3p targets two proteins important for myofiber assembly and protein–protein interaction, namely SORBS2 (SH3 domain-containing protein 2) and PDLIM5 (PDLIM5PDZ and LIM domain 5). Silencing of these two proteins drives CMCs to hypertrophy, while the inhibition of miR-21-3p attenuated the pathology [46]. RNA-sequencing studies from exosomes released from hypoxic CMCs identified the upregulation of the lncRNA AK139128. Hypoxic CMCs, treated with a siRNA against AK139128, in co-culture with fibroblasts, show that the uptake of exosomes stimulates fibroblast apoptosis and inhibits proliferation, migration, and invasion to a higher degree in the presence of AK139128. Moreover, exosomes from rat CMCs treated with siRNA against AK139128 or the scrambled control were administered to the infarct area of a rat model of MI: AK139128 expression levels were higher only for the control-treated rats, enhancing apoptosis, and reducing cell migration, thus exacerbating MI in rats, and providing a new layer of understanding of the mechanisms underlying hypoxia-related cardiac phenotypes [47].

Macrophages also regulate fibroblast activation and proliferation by secreting TGF-β, IL-6 (transforming growth factor-β and interleukin-6) and ncRNA-enriched EVs [55]. For example, *circUbe3* targets the miR-138-5p/RhoC axis, promoting fibrosis after MI. In detail, *circUbe3* acts as sponge for the miR-138-5p, inhibiting its activity, and in turn, rescuing the inhibition of RhoC by miR-138-5p and promoting cell proliferation. This provides evidence that EVs containing *circUbe3* exacerbate fibrosis after acute MI [48]. Therefore, targeting the release of this lncRNA into extracellular vesicles after MI could provide a method for limiting the fibrotic response.

Another example of a sponge circRNA is *circHIPK3*. Hypoxic CMCs secrete *circHIPK3* which becomes shuttled to endothelial cells. *circHIPK3* suppresses miR-29a activity in endothelial cells which leads to IGF-1 (insulin growth factor-1) increase [49]. IGF-1 is known to have anti-apoptotic effects by activating the p53-signaling pathway in cancer cells [56], and by activating the AKT/secreted frizzled-related protein 2 (SFRP2)/β-catenin pathway, protecting CMCs against apoptosis [57]. In endothelial cells exposed to oxidative stress, gain- and loss-of-function studies demonstrated that miR29a upregulates apoptosis and ROS levels through IGF-1 interaction, thus increasing endothelial dysfunction. In vivo, mice treated with exosomal *circHIPK3* derived from hypoxic CMCs are protected from oxidative injury via miR-29a/IGF-1 in cardiac microvascular endothelial cells [49].

Finally, miR-30d is transcribed and released into the bloodstream by CMCs under mechanical stress. Plasma levels of miR-30d were lower in patients with echocardiographic improvements under clinical cardiac resynchronization therapy. Functionally, miR-30d protects from hypertrophy in a canine model of HF by inhibiting deleterious TNF-α signaling [50]. This provides yet another example of an ncRNA which is necessary for the maintenance of cardiac homeostasis via cell-to-cell communication; however, little is still known in this context.

### 3.2. ncRNAs Mediating Cell Communication in Myocardial Infarct

The lncRNA *H19* is known as a fetal lncRNA, whose expression diminishes after birth [58,59]. Recent findings revealed that *H19* is upregulated in fibroblasts within the infarcted area of the heart in a murine model of MI. The infarct area accumulates activated fibroblasts that produce the excessive release of extracellular matrix (ECM) components, including Col1a1 (collagen type I) to preserve heart structure. In this context, a new role of the lncRNA *H19* was associated with ECM component release. Gain- and loss-of-function studies demonstrated that *H19* exacerbates fibrosis after injury [51]. Chromatin isolation by RNA purification (ChIRP) followed by mass spectrometry revealed that *H19* interacts with YB-1 (Y-box-binding protein 1) in NIH3T3 fibroblasts and hIPS-fibroblasts. YB-1 is an RNA- and DNA-binding protein that modulates gene expression both at the transcriptional and translational levels. In addition, it is strongly linked to fibrosis via Col1a1 regulation [60]. YB-1 suppresses Col1a1 expression; however, upon MI stress, the *H19*-YB-1 complex is established and expression of Col1a1 increases, promoting cardiac remodeling. Thus, *H19* inhibition in FBs is a promising therapeutic approach, as it prevents ECM deposition and cardiac remodeling after cardiac injury. Nonetheless, the exact mechanism of function of *H19* is not fully understood in the cardiovascular field. Another study revealed a cardioprotective role for *H19* against cardiac hypertrophy. RNA-immunoprecipitation experiments confirmed the interaction between *H19* and PRC2 (polycomb repressive complex 2) in cardiomyocytes. PRC2 mediates H3K27me3 (tri-methylation of the lysine residue 27 of Histone 3), a well-known histone mark of transcriptional repression. ChIP-sequencing experiments in HL-1 cells after the silencing of *H19* showed a global increase in H3K27me3 together with high methylation enrichment in the locus of *Tescalcin* (*Tesc*). *Tesc* is an NFAT mediator, which in turn, reduces its activity. Thus, *H19* prevents the PRC2-mediated epigenetic repression (via H3K27me3) of the *Tesc* locus, thus repressing NFAT signaling. Additionally, in vivo experiments in murine, porcine, and human engineered heart tissue (hEHT) models of cardiac hypertrophy demonstrated the anti-hypertrophic benefits of *H19* [52]. The dual role of *H19* as both cardio-protective or cardio-detrimental against cardiovascular disease can be explained by the fact that lncRNAs have different expression patterns and functions within each cell type, and can therefore be exploited as a therapeutic molecule by targeting a specific cell population.

Another cellular event that is involved in the onset of CVD, linked to cardiac remodeling, is autophagy, an intracellular process by which proteins and organelles are degraded to maintain cellular homeostasis. This process has been shown to be augmented in various CVDs, such as dilated cardiomyopathy, ischemic heart disease, and heart failure [61]. Whether autophagy is beneficial or harmful for the diseased heart is a matter of intense debate. It appears to have different roles depending on the origin of the stress and the signaling pathways that are dysregulated [62]. The novel lncRNA cardiac autophagy inhibitory factor (*CAIF*) was recently discovered as playing an important role in autophagy regulatory pathways. This lncRNA is an epigenetic regulator that blocks the binding of transcription factor p53 to myocardin, which has been linked to the autophagic response [63]. This inhibition of the p53–myocardin interaction by *CAIF* has been shown to inhibit autophagy and cell death in response to H_2_O_2_ in CMCs [53].

## 4. Cardiac Ageing

Age-related diseases are the new healthcare challenge worldwide due to the increase in lifespan in the last century [64]. Specifically, cardiac ageing is one of the principal risk factors for CVD [65]. Cardiac ageing is characterized by a decline in systolic and diastolic function, reduced myocardial contractility, and hypertrophy leading to HF, which is the major cause of death in the elderly [66]. Such cardiac remodeling highly affects cardiomyocyte structure and metabolism such as telomere shortening [67] and a metabolic switch from fatty acid oxidation to glycolysis [68]. In addition, ageing promotes endothelial cell senescence and inflammation as a result of oxidative stress or mitochondrial dysfunction [69]. How ageing affects such remodeling still remains largely unknown, and little is known about the signaling pathways linking oxidative stress, inflammation, and cellular communication with ageing. As with many other CVDs, there is an evident lack of pharmacological intervention able to reverse cardiac ageing; thus, it is of great importance to discover novel and targetable ncRNAs that regulate the physiological development of ageing and exert a block on cellular senescence.

### 4.1. ncRNA Mediating Cell Communication in Ageing

It was recently discovered that mesenchymal stem cell (MSC)-derived exosomes could prevent ageing-induced cardiac dysfunction. These exosomes are enriched for the lncRNA *MALAT-1* (metastasis-associated lung adenocarcinoma transcript 1). CMCs uptake *MALAT-1*-containing exosomes to downregulate the NF-*κ*B/TNF-α pathway. This pathway is activated when cultured CMCs are exposed to a cellular senescence stimulus (such as H_2_O_2_), an effect which is reduced when the cells are cultured in the presence of MSC-exosomes containing *MALAT-1*. *In vivo, MALAT-1* can delay the ageing process in the D-gal-induced animal ageing model and prevent ageing-induced cardiac dysfunction [70].

The anthracycline antibiotic doxorubicin (Dox) is an effective treatment for cancer, however, it is cardiotoxic, and consequently nearly 30% of patients suffer from cardiovascular complications after the treatment [71]. In cultured CMCs, Dox induces senescence by damaging the DNA. In this context, a novel role for the previously mentioned lncRNA *NEAT-1* was described. It has been previously reported that the Migration Inhibitory Factor (MIF) promotes MSC vesiculation, and that MSC-derived exosomes have cardioprotective potential against ischemic stress [72,73]. Senescent CMCs were therefore incubated with MSC-MIF exosomes containing *NEAT-1*. Upon *NEAT-1* uptake, they observed an inhibition of miR-221-3p that alleviated Dox-induced cardiac injury and recovered telomere length and activity. More specifically, *NEAT-1* acts as ceRNA, sponging miR-221-3p activity and limiting its functionality [74]. In this context, Sirt2 was identified as a potential target against the anti-senescent effect of miR-221-3p. Sirt2 deficiency was reported to promote genetic instability, which is a prevalent event occurring during cellular senescence [75]. This study revealed a stem-cell based therapy against cellular senescence based on the newly described exosome/lncRNA–*NEAT1*/miR-221-3p/Sirt2 pathway [74], providing a prominent example of ncRNAs transported for cell–cell communication [76].

### 4.2. ncRNA Dysregulated in the Course of Ageing

A novel lncRNA, *SARRAH* (SCOT1-antisense RNA regulated during ageing in the heart), was recently described in human engineered heart tissue (EHT). *SARRAH* is downregulated in heart tissue in the elderly and infarcted hearts, and interestingly, GapmeR knockdown of *SARRAH* induces apoptosis and delays cardiac contractile force. Mechanistically, this lncRNA allows the transcription of cardiac survival genes by forming a DNA-DNA-RNA triplex and by recruiting two transcription factors, namely CRIP2 and p300. P300 is an acetyltransferase that acetylates histone H3 on lysine 27 (H3K27ac) to activate transcription: therefore, the lncRNA *SARRAH* has been associated with open (active) chromatin states. On the other hand, an improvement in cardiac function was observed when *SARRAH* was overexpressed, using AVV9 in a mouse model of MI. This study demonstrates the potency of lncRNAs as epigenetic modulators of the chromatin state and for recruiting transcriptions factors, suggesting a new epigenetic pathway that can be used to treat CVD in the elderly [77].

Finally, *Meg3* is a highly expressed lncRNA in senescent HUVEC cells and also in human cardiac atria from the elderly. *Meg3* interacts with Ezh2 and Jarid2 (factors of the PRC2 complex previously described) to epigenetically mediate changes to gene expression, inducing the silencing of genes related to EC differentiation. The lncRNA Meg3 facilitates the JARID2–PRC2 interaction on chromatin, suggesting a mechanism by which lncRNAs contribute to PRC2 recruitment [78]. Furthermore, GapmeR knockdown of *Meg3* improved endothelial cell function in vivo and in vitro, and resulted in a rescue of impaired endothelial cell function in old mice [79].

## 5. Conclusions and Future Perspectives

Being able to regulate gene expression both at the transcriptional and post-transcriptional levels, the dysregulation of ncRNAs can lead to the development of various pathologies, including cancer, neurological disorders, and cardiovascular diseases. For this reason, therapeutic approaches based on the regulation of specific ncRNAs are very promising strategies for the treatment of these pathologies [80,81,82].However, most current ncRNA-based drugs have two noteworthy limitations. Firstly, RNA molecules can be recognized by the immune system causing adverse immune reactions. Secondly, due to the ubiquitous prevalence of their targets, ncRNA-based drugs may not be site-specific [83]. In light of this, ncRNAs are increasingly proposed for therapeutic applications in association with biocompatible nanoparticles (NPs). These are small particles with a diameter ranging from 1 to 100 nm, already effectively used for the transport and release of antibiotics, anticancer drugs, and for viral or non-viral genes to the site of interest without being recognized by the immune system [84].

Due to their ability to act either as oncogenes or as tumor suppressors, miRNAs are among the most studied ncRNAs in the cancer field. In particular, miRNA mimic drugs are used to increase endogenous levels of tumor suppressor miRNAs, whereas anti-miRNA-based drugs are used to inhibit the action of specific oncogenic miRNAs. In order to increase specificity, and reduce the instability and toxicity of these drugs, their use in association with NPs is proving to be an effective choice [85]. For example, a recent study showed that mPEG-PLGA-PLL (PEAL) NPs loaded with miR-99a were able to specifically inhibit tumor xenograft growth in hepatic carcinoma (HCC)-bearing mice. The specific and controlled release of this miRNA at the tumor level was obtained using PEAL NP functionalization with both LA and VEGFab. The former specifically binds the asialoglycoprotein receptor, which is highly expressed on the surface of HCC cells, whereas the latter binds VEGF, which is an endothelial marker abundantly expressed in HCC [86]. It has also been shown that PLGA nanoparticles modified with penetratin, a cell-penetrating peptide, are able to induce an optimized release of anti-miR-155 molecules into an in vivo model of lymphoma. This release results in the inhibition of the oncogenic miR-155 and in the reduction in tumor growth over the course of 4–6 days after drug administration [87]. In addition to miRNAs, the use of siRNA-conjugated NPs capable of inhibiting the oncogenic activity of some lncRNAs is also increasingly widespread in the field of cancer. For example, lipid NPs loaded with si-LINC01257 were recently used to silence the enhanced expression of LINC01257, typical of children with acute myeloid leukemia, in t [8,21] Kasumi-1 AML cells [88]. Furthermore, in order to silence the oncogenic activity of CCAT1 lncRNA, siCCAT1 was conjugated to polymeric hybrid nanoparticles (CSNP). In this way, it was possible to obtain a good anti-tumor effect in the HT-29 subcutaneous xenograft model of colorectal cancer [89]. Finally, by exploiting the enhanced permeability and retention (EPR) effect, Bi et al. were able to deliver NPs loaded with siAFAP1-AS1 to the tumor site. This siRNA reduced the levels of AFAP1-AS1 lncRNA, improving the efficacy of radiotherapy in both xenograft and metastatic triple-negative breast cancer models [90].

In the cardiovascular field, many ncRNAs have been shown to be involved in the development of diseases such as atherosclerosis, myocardial infarction, and heart failure [91,92,93,94,95]. However, the major limitation to date is the use of ncRNA-based therapies, and more specifically, very few have been proposed as strategies for targeted drug delivery in association with nanoparticles. Interestingly, most of the studies carried out to date in this perspective consider the use of miRNA mimics or anti-miRNA-based drugs. For example, biocompatible and biodegradable calcium phosphate (CaP) nanoparticles have recently been proposed as an effective system capable of delivering miR-133 exclusively to the heart. The downregulation of this miRNA within cardiomyocytes is, in fact, involved in the development of a hypertrophic phenotype. The cardiac specificity of these nanoparticles lies in their negative surface charge conferred on them by citrate, which makes them more inclined to polarized cells such as cardiomyocytes [96]. For the treatment of atherosclerosis, the use of coated cationic lipoparticles (CCLs) has been proposed to deliver an anti-miR-712 at the level of the inflamed endothelial region. In the presence of a disturbed blood flow (d-flow), endothelial cells tend to express more miR-712, a pro-atherogenic mRNA involved in the formation of atherosclerotic plaques. In order to deliver the anti-miR-712 drug exclusively to the level of these cells, Kheirolomoom et al. conjugated the nanoparticles with VHPK, a peptide capable of binding the vascular cell adhesion molecule 1 (VCAM1) expressed on the surface of the inflamed endothelial cells. In this way, the authors were able to inhibit atherosclerosis in ApoE^-/-^ mice, avoiding the toxic systemic effects that the administration of naked anti-miR-712 alone would have generated [97]. Instead, in order to prevent the onset of heart failure following myocardial infarction, the use of nanoparticles conjugated with miR-155-5p as a complement to reperfusion has recently been proposed. This miRNA is able to act as a vasculoprotector during inflammatory processes, silencing the BACH1 mRNA in endothelial cells. To deliver miR155-5p exclusively to the level of endothelial cells in the infarcted cardiac region. Antunes et al. developed polymer nanoparticles composed of a hydrophobic core of PIBCA and a hydrophilic shell consisting of fucoidan, dextran, and DEAE-dextran. By means of DEAE-dextran, they managed to electrostatically bind miR-155-5p to the nanoparticles, exploiting the high affinity of fucoidan for P-selectin, a molecule highly expressed on the membrane of endothelial cells in the infarcted regions, thus managing to convey selectively miR-155-5p to activated hCAEC cells. In this way, they were able to reduce the expression of BACH1 obtaining a targeted cytoprotective effect. The authors therefore proposed this therapeutic approach as a potential strategy to reduce the cellular damage that occurs in the infarcted heart [98]. Furthermore, following myocardial infarction, an excessive ROS production is activated in macrophages. This process is driven by the overexpression of *Nox2*, which further contributes to the deterioration of cardiac function [99]. This prompted Somasuntharam et al. to develop acid-degradable polyketal nanoparticles capable of delivering Nox2-siRNA to the infarcted heart. The intramyocardial administration of this ncRNA in C57BL/6 mice was, in fact, effective both in preventing the upregulation of Nox2 within macrophages and in attenuating cardiac dysfunction [100]. Interestingly, a few years later, these authors used the same polyketal nanoparticles for the administration of three different miRNAs (miR-106b, miR148b, miR-204) capable of regulating the expression of Nox2. Again, miRNA-based therapy delivered by means of nanoparticles was found to be effective in improving cardiac function in vivo by significantly reducing Nox2 levels [101]. Finally, as far as we know, only a recent study has proposed the use of siRNA-conjugated nanoparticles able to interfere with lncRNAs involved in the development of cardiovascular disease. By means of neutrophil membrane (NM)-camouflaged mesoporous silica nanocomplex (MSN), Shi et al. were able to inhibit ferroptosis by silencing the activity of the lncRNA AAB, which was upregulated during cardiac hypertrophy as a result, specifically in cardiac microvascular endothelial cells (CMECs). The targeted delivery of these si-AAB nanoparticles in vivo was obtained through the binding of the antibody LFA-1 and β2 Integrin to ICAM-1, a protein highly expressed in the endothelial cells of hypertrophic hearts [102].

The use of functionalized nanoparticles for the administration of ncRNA-based drugs exclusively in diseased cells is therefore a promising strategy which allows for a significant reduction in the amount of drug to be administered, its toxicity, and its side effects, thus increasing its safety. Moreover, by preventing ncRNA-based drugs from being recognized by the immune system, NPs also help to improve their stability, bioavailability, and pharmacokinetics. The use of nanoparticles as drug delivery “vehicles” in the context of cardiovascular disease mimics the physiological release of ncRNAs through extracellular vesicles in order to maintain cardiac homeostasis, and should further be exploited as potential therapeutic targeting mechanisms. However, despite the promising results obtained to date in the cardiovascular field, further investigation is needed in order to use biocompatible nanoparticles as safe and efficient delivery systems for ncRNA-based drugs.

## Figures and Tables

**Table 1:** ncRNA in Atherosclerosis.

ncRNA	Target	Function	Cell Type/Communication	Ref.
miR-143/145	*HKII* and *ITGβ8*	VSMC differentiation of EC’s response to shear stress	VSMC–EC, tunneling nanotubes	[9]
*RNCR3*	*KLF2*	ceRNA of miR-185-5p. Confers vasoprotection to ECs	EC–VSMC Exosomes	[12]
miR-92	*KLF4*	Modulates macrophage plasticity	EC–Macrophages EVs	[13]
miR-155	NF-κB	Implicated in vascular inflammatory response	Neutrophils–EC MVs	[14]
*Circ_Lpr6*	miR-145	Sponges miR-145, affects migration, differentiation, and proliferation of VSMC	VSMC	[15]
miR-10a	GATA6/VCAM-1	EC: inhibits vascular cell adhesion. Leukocytes: reduces infiltration	EC, Leukocytes	[16]
*SNHG12*	DNA-PK and Ku70/Ku80	Inhibits interaction between DNA-PK and Ku70/Ku80, exacerbating DNA damage	EC	[17]
*NEXN-AS1*	BAZ1A and *NEXN*	*NEXN-AS1* reduces BAZ1A activity to relax chromatin and allow *NEXN* expression	EC	[18]
MIAT	ERK/ELK1/EGR1 pathway	Reduces proliferation and increase apoptosis	EC	[19]

**Table 2:** ncRNA in pathological cardiac remodeling.

ncRNA	Target	Function	Cell Type/Communication	Ref.
ENSMUST00000122745	Unknown	Unknown	CMC–FB large EVs	[45]
Neat1	P53	Promotes cellular survival	CMC–FB Small EVs	[45]
miR-21-3p	SORBS2 and PDLIM5	Aggravates hypertrophy in CM	FB-CM Exosomes	[46]
AK139128	Unknown	Inhibits proliferation, migration, and invasion	CM-FB Exosomes	[47]
Circ_Ube3	miR-138-5p	Inhibits miR-138-5p to express RhoC, exacerbating fibrosis.	Macrophages-FB EVs	[48]
circHIPK3	miR-29a	Protects from oxidative stress via miR-29a/IGF-1	CM-EC Exosomes	[49]
miR-30d	TNF-α signalling	Protects from Hypertrophy	CM-plasma Exosomes	[50]
H19	FB: YB-1 CM: PRC2	FB: Promotes fibrosis and release of ECM components (Col1a1) CM: Anti hypertrophic via H3K27me3	CMC, FB	[51,52]
CAIF	P53	Blocks the binding of the transcription factor p53 to myocardin inhibiting autophagy	CM	[53]

## Data Availability

Not applicable.
